# Exosomal miRNA-205 promotes breast cancer chemoresistance and tumorigenesis through E2F1

**DOI:** 10.18632/aging.203298

**Published:** 2021-07-22

**Authors:** Yan Zhao, Li-Jun Jin, Xiao-Yu Zhang

**Affiliations:** 1 Thyroid and Breast Department, Extra-Thyroid and Breast Neoplasms, Cangzhou Central Hospital, Cangzhou, Hebei, China; 2 Department of Thyroid and Breast III, Cangzhou Central Hospital, Cangzhou, Hebei, China

**Keywords:** breast cancer, exosome, chemoresistance, tumorigenesis, miRNA-205

## Abstract

Breast cancer (BC) is a common malignant tumor in females. The challenge in treating BC is overcoming chemoresistance. Exosome-mediated transfer of miRNAs is a molecule-shuttle in intercellular communication. Thus, we aimed to investigate whether exosomal miRNA-205 could affect chemoresistance and tumorigenesis in recipient tumor cells and to elucidate the underlying mechanism *in vivo* and *in vitro*. Microarray and qRT-PCR assays demonstrated that miRNA-205 was upregulated in tamoxifen resistance MCF-7/TAMR-1 (M/T) cells and M/T cell-derived exosomes (M/T-Exo). The M/T-Exo was internalized by human BC cells (BCCs), causing increased expression of miRNA-205 in BCCs. Coculturing with M/T-Exo promoted tamoxifen resistance, proliferation, migration, and invasion while suppressed apoptosis in recipient BCCs, which were associated with activating the caspase pathway and phosphorylating Akt. Luciferase reporter assays showed that miRNA-205 directly targeted E2F Transcription Factor 1 (E2F1) in BCCs. Furthermore, knockdown of miRNA-205 or overexpression of E2F1 reversed the roles of M/T-Exo in BCCs. *In vivo* experiments showed that the intratumoral injection of M/T-Exo caused greater tamoxifen resistance and larger tumor size relative to mice treated with miRNA-205-knockdown or E2F1-overexpressing BCCs. Together, the results suggest that exosomal miRNA-205 may promote tamoxifen resistance and tumorigenesis in BC through targeting E2F1 *in vivo* and *in vitro*.

## INTRODUCTION

Breast cancer (BC) is the most common invasive malignancy among women worldwide, accounting for 31% of all female cancer types [1]. Over the past two decades, the incidence of BC has nearly doubled [2]. Currently, effective therapeutic strategies for BC patients include surgery, chemotherapy, hormonal manipulation, targeted treatment, radiotherapy, or a combination thereof [3]. However, statistical evidence has revealed that the five-year survival rate of patients with BC is still relatively low, although advancements in early diagnosis and therapy have been made during the past decades [4]. Thus, it is urgent to investigate new treatments for BC.

Although the initial treatment has been demonstrated to be effective for most patients with BC, eventually, more aggressive tumors develop due to the resistance of tumor cells from radiotherapy or chemotherapy [5, 6]. Thus, metastasized recurrence causing a poor prognosis has become the major challenge and obstacle for developing BC therapeutics. On the other hand, tamoxifen, an antagonist of the estrogen receptor (ER), is widely applied to treat ER-positive BC [7]. In spite of advances in diagnosis and treatment of BC, it has been reported that BC patients treated with tamoxifen relapse [8, 9]. This suggests that the mechanism of tamoxifen resistance is still not fully understood. As such, understanding the molecular pathways behind tamoxifen resistance would greatly contribute to the development of high-sensitivity therapies for BC.

Exosomes are a group of extracellular vesicles (30–150 nm in size) [10, 11]. Growing studies reported that exosomes are capable of transferring functional molecules, including mRNAs, miRNAs, enzymes, and lipids, to neighboring or distant cells, eventually influencing their cellular activities [12]. Thus, exosomes have been regarded as an ideal molecule-shuttle in intercellular communication. Similar to other cell types [12], cancer cells can also secrete exosomes containing functioning molecules that are associated with various cancer-related activities, such as angiogenesis [13, 14], metastasis [15, 16], progression [17] and chemoresistance [18].

MicroRNAs (miRNAs) are a class of small (21–24 nucleotides in length) non-coding RNAs [19]. The miRNAs function as an essential regulatory mechanism of gene expression, primarily through binding target mRNAs and eventually silencing mRNAs or promoting mRNA degradation [20]. Given such important roles, miRNAs are a critical regulator in various physiological and pathological processes [21], such as chemoresistance [22, 23]. As a multifunctional factor, miRNA-205 is essential for BC cell metastasis, stemness, and epithelial-mesenchymal transition (EMT) [24] and is a potential diagnostic marker for the early detection of BC [25]. Also, the involvement of miRNA-205 in chemoresistance has been reported in several cancers, such as BC [26], pancreatic cancer [27], hepatocellular carcinoma [28], and non-small cell lung cancer [29]. However, the effect of exosomal miRNA-205 on tamoxifen chemoresistance in BC remains to be elucidated.

E2F Transcription Factor 1 (E2F1), a transcription activator, can bind to DNA with dimerization partner (DP) proteins through the recognition site of E2 [30]. It has been demonstrated that the dissociation of E2F1 from retinoblastoma protein can restore the transcriptional function of E2F1, which is a major driving force for the cell cycle [31]. Given its critical roles in cellular activities, numerous studies report that E2F1 participates in chemoresistance, metastasis, and progression in several cancers [32, 33]. For BC, Hollern et al. report that E2F1 promotes BC metastasis through gene fibroblast growth factor 13 (Fgf13) [34]. Also, miRNA-93-induced chemosensitivity to paclitaxel is mediated by E2F1 and Cyclin D1 in BC [35].

Therefore, this study aimed to determine whether miRNA-205 can be transmitted via exosomes derived from chemoresistant breast cancer cells (BCCs) and the roles of exosomal miRNA-205 in the regulation of tamoxifen chemoresistance and tumorigenesis in BC *in vitro* and *in vivo*.

## RESULTS

### Isolation and identification of exosomes derived from BCCs and M/T cells

**Figure 1:**
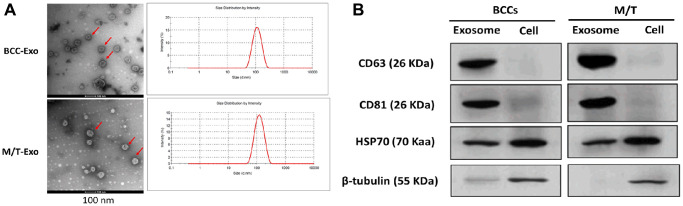
**Features of exosomes isolated from breast cancer cells (BCCs) and MCF-7/TAMR-1(M/T) cells.** (**A**) Representative images of exosomes isolated from BCCs and M/T cells, as photographed using the transmission electron microscope and the range of exosome diameter (right column). Scale bar = 500 nm (left column) and 200 nm (middle column). (**B**) The expressions of exosome markers in BCCs and M/T cells and their exosomes, as detected with western blots.

Exosomes were first isolated from BCCs and M/T cells. The morphology of exosomes was identified by the transmission electron microscope which showed round-shape exosomes with bilayer membranes (Figure 1A). Also, the exosome size distribution revealed that the diameter ranged from 40 to 140 nm and the predominant size of exosomes was 120 nm (Figure 1A). Next, both exosomes derived from BCCs and M/T cells positively expressed the exosomal markers, CD63, CD81, and HSP70, while β-tubulin was positively expressed in the BCCs and M/T cell lysates (Figure 1B). Together, these results suggest that the exosomes isolated from BCCs and M/T cells displayed typical features of exosomes, which were then used in subsequent experiments.

### Upregulation of miRNA-205 in M/T cells and exosomes enhances tamoxifen resistance

**Figure 2:**
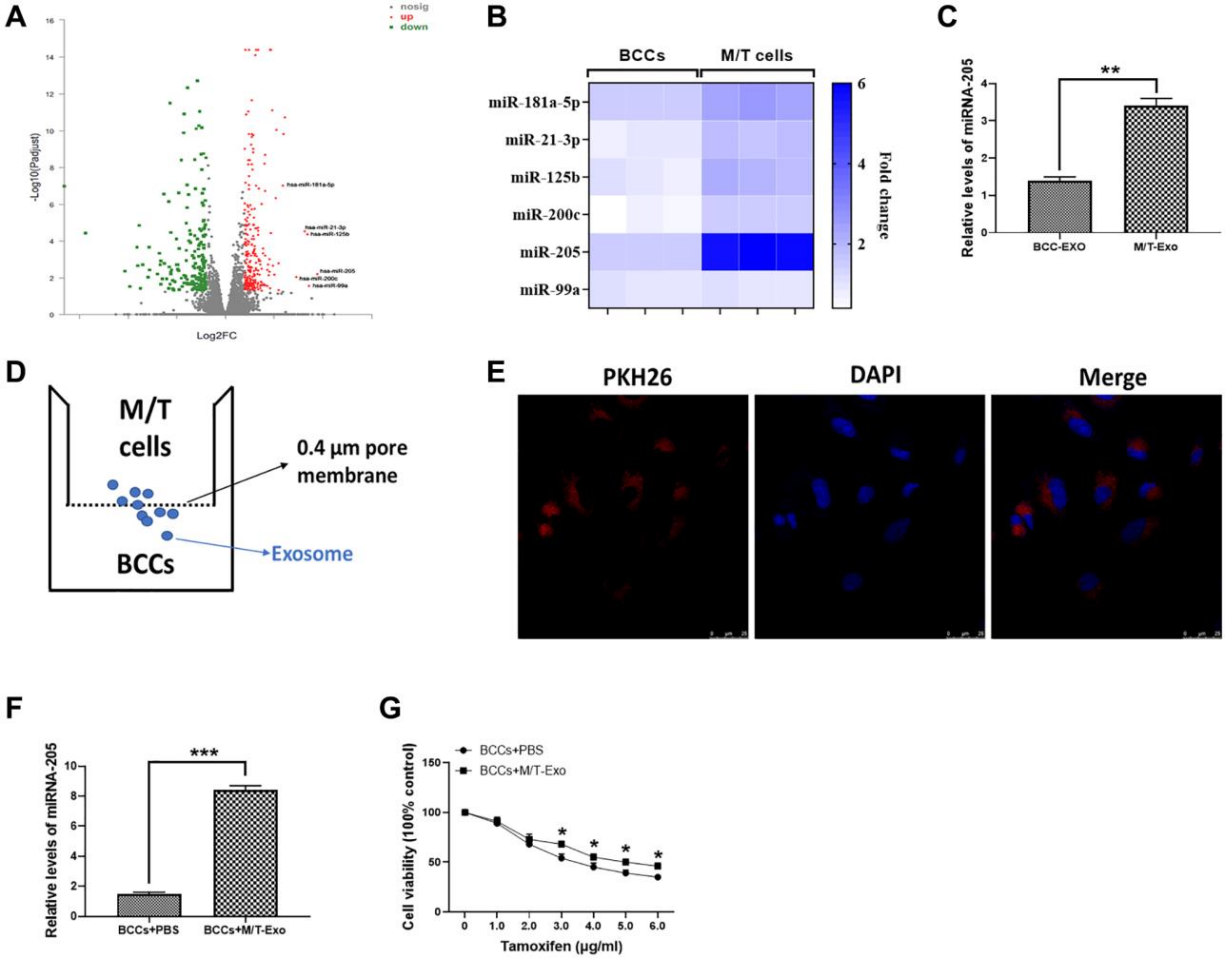
**Upregulation of miRNA-205 is associated with the tamoxifen resistance of breast cancer cells (BCCs).** (**A**) Volcano plot of miRNA profile in BCCs and M/T cells, as determined by microarray analysis. (**B**) The expressions of miRNAs in BCCs and M/T cells, as shown through the heat map. (**C**) The expression of miRNA-205 in exosomes isolated from BCCs and M/T cells. (**D**) Graphic illustration of the coculture system. The M/T cells (upper chamber) and BCCs (lower chamber) were separated by a 0.4 μm pore membrane, which only allows the passage of exosomes, but not large molecules. (**E**) PKH26-labeled (red fluorescence) M/T exosomes were taken up by DAPI-stained BCCs (blue fluorescence), as photographed using the confocal microscopy. Scale bar = 25 μm. (**F**) The expression of miRNA-205 in BCCs treated with PBS or M/T-Exo. (**G**) Cell viability of BCCs treated with PBS or M/T-Exo. Values are means ± SD. ^*^P < 0.05; ^**^P < 0.01, ^***^P < 0.001. At least three replicates were available for analysis in each treatment group.

The microarray assay was used to determine the miRNA expression profile in M/T cells. As shown in Figure 2A, a group of miRNAs was found to be upregulated in M/T cells, compared with BCCs. Among these upregulated miRNAs, including miR-181a-5p, miR-21-3p, miR-125b, miR-200c, miR-205, and miR-99a, we next performed qRT-PCR to verify their expression. The results showed that miRNA-205 displayed the greatest increased trend in M/T cells than those of BCCs (Figure 2B). Therefore, we focused our attention on the role of miRNA-205 during BC chemoresistance. In this study, the expression of miRNA-205 was significantly higher in exosomes derived from M/T cells than those of BCCs (Figure 2C), indicating the potential role of miRNA-205 in chemoresistance of BC. Therefore, the hypothesis was that exosomal miRNA-205 from M/T cells may influence tamoxifen resistance of BCCs. To test this hypothesis, a coculture system was applied to deliver exosomes derived from M/T cells (M/T-Exo) to BCCs (Figure 2D). To visualize the exosomal transfer, PKH26, a fluorescent tracer, was applied to label M/T-Exo. After incubation, the red fluorescence in the cytoplasm of BCCs through confocal microscope was detected (Figure 2E). Meanwhile, the expression of miRNA-205 was increased in BCCs cocultured with M/T-Exo than those treated with PBS (Figure 2F), suggesting that the upregulation of miRNA-205 may be transferred from M/T cells to BCCs via M/T-Exo. Next, to investigate the role of M/T-Exo in tamoxifen resistance of BCCs, tamoxifen was applied to treat BCCs cocultured with M/T-Exo. The results assessed by CCK-8 revealed that the cell viability of BCCs cocultured with M/T-Exo was higher than those cocultured with PBS (Figure 2G), indicating that M/T-Exo may promote tamoxifen resistance in BCCs, which may be associated with exosomal miRNA-205 transfer.

### E2F1 is a direct target of miRNA-205 and involved in M/T-Exo-induced tamoxifen resistance in BCCs

**Figure 3:**
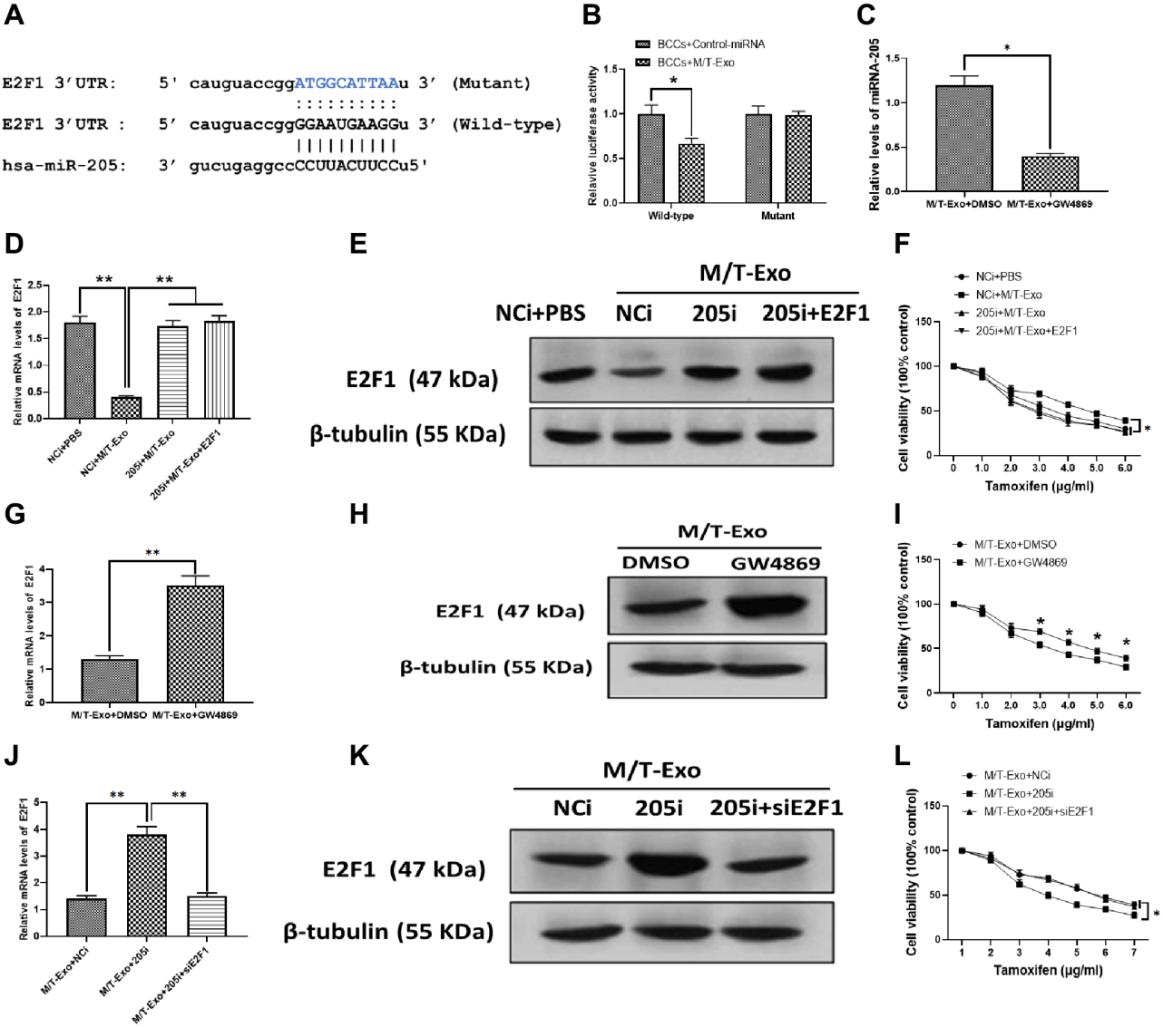
**M/T-Exo miRNA-205 enhances the tamoxifen resistance of breast cancer cells (BCCs) via targeting E2F1.** (**A**) The putative sequence of miRNA-205 binding sites in the 3′UTR of E2F1. (**B**) Relative luciferase activity. (**C**) The expression of miRNA-205 in M/T-Exo-cocultured BCCs treated with DMSO or GW4869. (**D**–**E**) The mRNA and protein expressions of E2F1 in M/T-Exo-cocultured BCCs treated with the negative control miRNA-205 inhibitor (NCi), miRNA-205 inhibitor (205i), or the combination of 205i and lentiviral vector carrying E2F1 (E2F1). (**F**) Cell viability of M/T-Exo-cocultured BCCs treated with the negative control miRNA-205 inhibitor (NCi), miRNA-205 inhibitor (205i), or the combination of 205i and lentiviral vector carrying E2F1 (E2F1). (**G**–**H**) The mRNA and protein expressions of E2F1 in M/T-Exo-cocultured BCCs treated with DMSO or GW4869. (**I**) Cell viability of M/T-Exo-cocultured BCCs treated with DMSO or GW4869. (**J**–**K**) The mRNA and protein expressions of E2F1 in M/T-Exo-cocultured BCCs treated with the negative control miRNA-205 inhibitor (NCi), miRNA-205 inhibitor (205i), or the combination of 205i and E2F1 siRNA (siE2F1). (**L**) Cell viability of M/T-Exo-cocultured BCCs treated with the negative control miRNA-205 inhibitor (NCi), miRNA-205 inhibitor (205i), or the combination of 205i and E2F1 siRNA (siE2F1). Values are means ± SD. ^*^P < 0.05, ^**^P < 0.01, ^***^P < 0.001. At least three replicates were available for analysis in each treatment group.

Thus, to further investigate the molecular mechanism underlying exosomal miRNA-205-associated tamoxifen resistance in BCCs, bioinformatics tools, TargetScan [36] and Starbase [37], were employed to predict the target gene of miRNA-205 in BCCs. The results showed that E2F1 contained a putative binding site in 3′UTR of miRNA-205 (Figure 3A). The luciferase reporter assays were performed to verify this prediction and showed the M/T-Exo reduced the relative luciferase activity in BCCs transfected with the wild-type E2F1 3′UTR, whereas no impact on luciferase activity was found in BCCs treated with the mutant E2F1 3′UTR (Figure 3B). To verify whether miRNA-205 is transferred through exosomes, GW4869 was applied to block the secretion of exosomes from M/T cells in the coculture system. After incubation, the expression level of miRNA-205 was significantly reduced in the BCCs cocultured with M/T cells and GW4869 compared with those cocultured with M/T cells without the GW4869 treatment (Figure 3C), indicating M/T-Exo may be an important shuttle to transfer miRNA-205 from M/T cells to BCCs.

### E2F1 participates in miRNA-205-associated tamoxifen resistance in BCCs

To investigate the functional correlation between miRNA-205 and E2F1 in tamoxifen resistance of BCCs, the BCCs were transfected with the miRNA-205 negative control inhibitor (NCi), miRNA-205 inhibitor (205i), or the combination of 205i and E2F1-expressing vector (E2F1). Next, the M/T-Exo with their respective treatments were added into the BCCs medium. The results showed that the mRNA and protein expressions of E2F1 decreased in M/T-Exo-treated BCCs transfected with NCi but was not affected in the other treatment groups (Figure 3D and 3E). Meanwhile, the cell viability of BCCs treated with NCi and M/T-Exo were higher than those in all other treatment groups, which suggests an increase in tamoxifen resistance (Figure 3F). These results indicate that M/T-Exo could inhibit the expression of E2F1 through upregulating miRNA-205. Moreover, to further determine the roles of M/T-Exo in the inhibitory effect of E2F1 in BCCs, GW4869 was applied to block the secretion of exosomes from M/T cells in the coculture system. The mRNA and protein expressions of E2F1 were evaluated, which showed that E2F1 expression was higher in BCCs cocultured with M/T cells that were treated with GW4869 compared to the control group (Figure 3G and 3H). Accordingly, BCCs cocultured with GW4869-treated M/T cells displayed reduced cell viability when exposed to tamoxifen (Figure 3I). Furthermore, to determine the role of miRNA-205 in the inhibitory effect of M/T-Exo on E2F1, either NCi, 205i, or the combination of 205i and E2F1 siRNA (siE2F1) were applied to treat M/T cells in the upper chamber of the coculture system. After incubation, the BCCs in the lower chamber had significantly higher mRNA and protein expressions of E2F1 in the 205i-treated group compared to the other two BCCs treatment groups (Figure 3J and 3K). Similarly, BCCs expressed higher levels of E2F1 and showed lower cell viability when exposed to tamoxifen compared to those treated with NCi or the combination of 205i and E2F2 siRNA (Figure 3L). Collectively, the results suggest that the inhibitory effect of M/T-Exo on E2F1 may be mediated by exosomal miRNA-205.

### Exosomal miRNA-205 inhibits apoptosis in BCCs via the caspase signaling pathway

**Figure 4:**
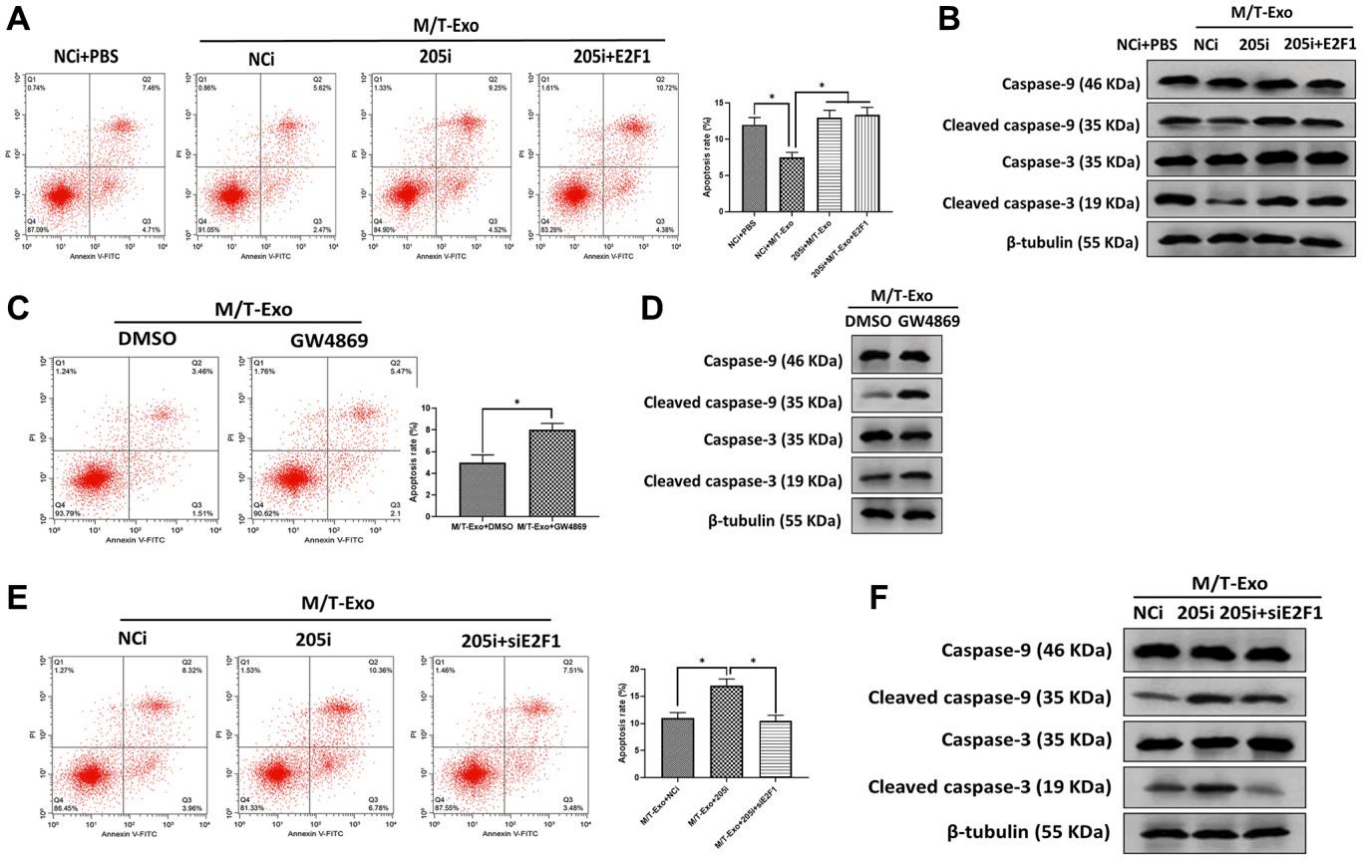
**M/T-Exo miRNA-205 inhibits breast cancer cells (BCCs) apoptosis via targeting E2F1.** (**A**) The apoptotic level of M/T-Exo-cocultured BCCs treated with the negative control miRNA-205 inhibitor (NCi), miRNA-205 inhibitor (205i), or the combination of 205i and lentiviral vector carrying E2F1 (E2F1). (**B**) The protein expressions of cleaved caspase-9 and caspase-3 in M/T-Exo-cocultured BCCs treated with the negative control miRNA-205 inhibitor (NCi), miRNA-205 inhibitor (205i), or the combination of 205i and lentiviral vector carrying E2F1 (E2F1). (**C**) The apoptotic level of M/T-Exo-cocultured BCCs treated with DMSO or GW4869. (**D**) The protein expressions of cleaved caspase-9 and caspase-3 in M/T-Exo-cocultured BCCs treated with DMSO or GW4869. (**E**) The apoptotic level of M/T-Exo-cocultured BCCs treated with the negative control miRNA-205 inhibitor (NCi), miRNA-205 inhibitor (205i), or the combination of 205i and E2F1 siRNA (siE2F1). (**F**) The protein expressions of cleaved caspase-9 and caspase-3 in M/T-Exo-cocultured BCCs treated with the negative control miRNA-205 inhibitor (NCi), miRNA-205 inhibitor (205i), or the combination of 205i and E2F1 siRNA (siE2F1). Values are means ± SD. ^*^P < 0.05, ^**^P < 0.01, ^***^P < 0.001. At least three replicates were available for analysis in each treatment group.

Next, M/T-Exo was applied to treat BCCs that were treated with NCi, 205i, or the combination of 205i and E2F1. Next, the apoptotic level was assessed by flow cytometry assays which demonstrated that BCCs treated with NCi and M/T-Exo exhibited lower apoptotic levels compared to the other groups (Figure 4A), along with decreased protein expressions of cleaved caspase-9 and caspase-3 (Figure 4B). Also, BCCs cocultured with M/T cells and GW4869 displayed higher levels of apoptosis than BCCs in the control group (Figure 4C), with enhanced protein expressions of cleaved caspase-9 and caspase-3 (Figure 4D). Moreover, the combination of M/T-Exo and 205i caused more apoptotic BCCs than those treated with the combination of M/T-Exo and NCi or the combination of M/T-Exo, 205i and siE2F1, with elevated levels of cleaved caspase-9 and caspase-3 (Figure 4E and 4F). Accordingly, the levels of cleaved caspase-9 and caspase-3 were also elevated using the combination of M/T-Exo plus 205i in BCCs. Together, exosomal miR-205 may promote tamoxifen resistance in BCCs via suppressing apoptosis through dampening the caspase signaling pathway.

### Exosomal miRNA-205 promotes cell proliferation, migration, and invasion in BCCs via the Akt signaling pathway

**Figure 5:**
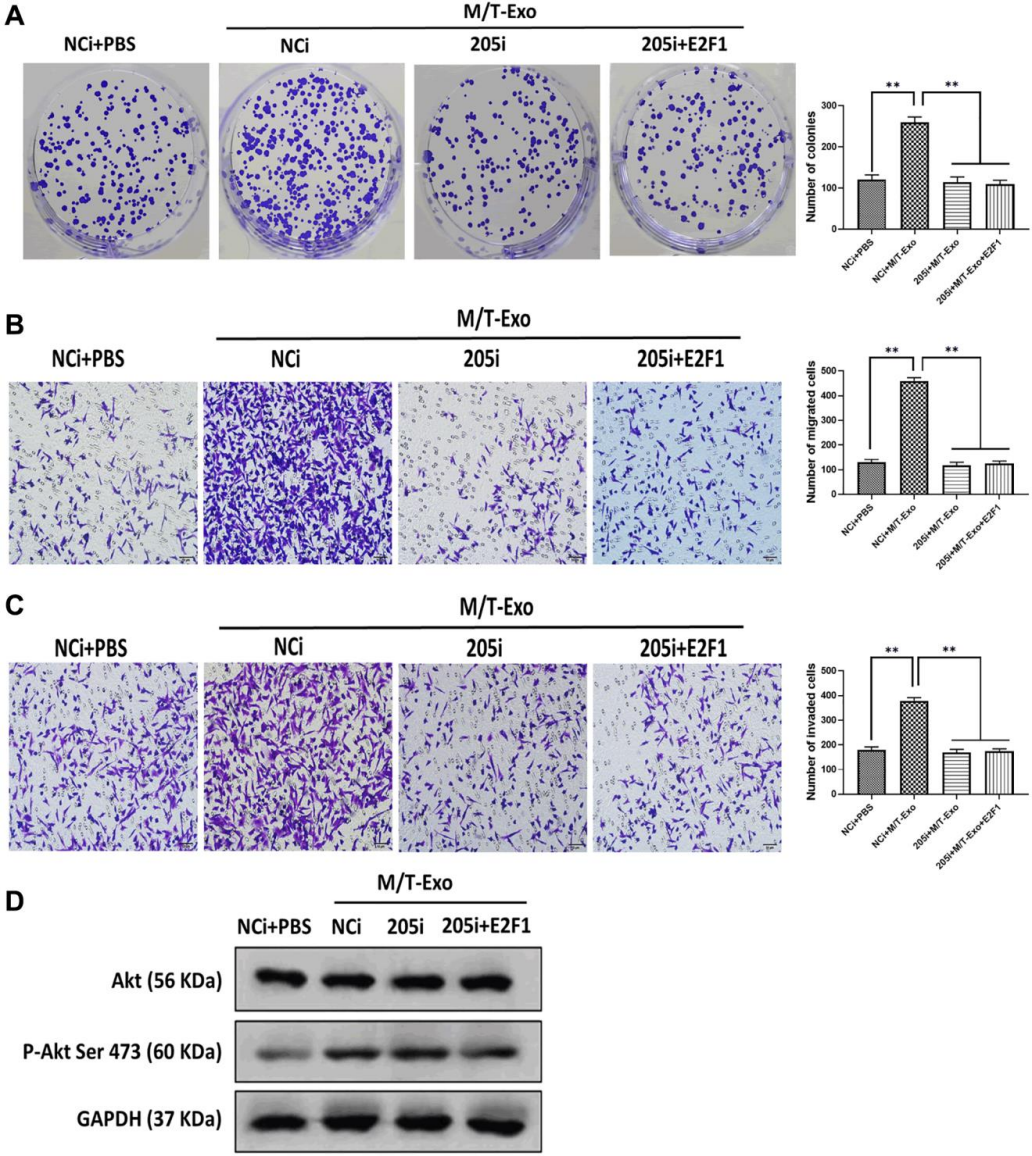
**M/T-Exo miRNA-205 affects breast cancer cells (BCCs) proliferation, migration, and invasion via targeting E2F1.** (**A**–**C**) The abilities of colony formation, migration, and invasion in M/T-Exo-cocultured BCCs treated with the negative control miRNA-205 inhibitor (NCi), miRNA-205 inhibitor (205i), or the combination of 205i and lentiviral vector carrying E2F1 (E2F1). (**D**) The protein expression of Akt and the phosphorylation of Akt at Ser 473 (p-Akt Ser 473) in M/T-Exo-cocultured BCCs treated with the negative control miRNA-205 inhibitor (NCi), miRNA-205 inhibitor (205i), or the combination of 205i and lentiviral vector carrying E2F1 (E2F1). Values are means ± SD. ^*^P < 0.05, ^**^P < 0.01, ^***^P < 0.001. At least three replicates were available for analysis in each treatment group.

**Figure 6:**
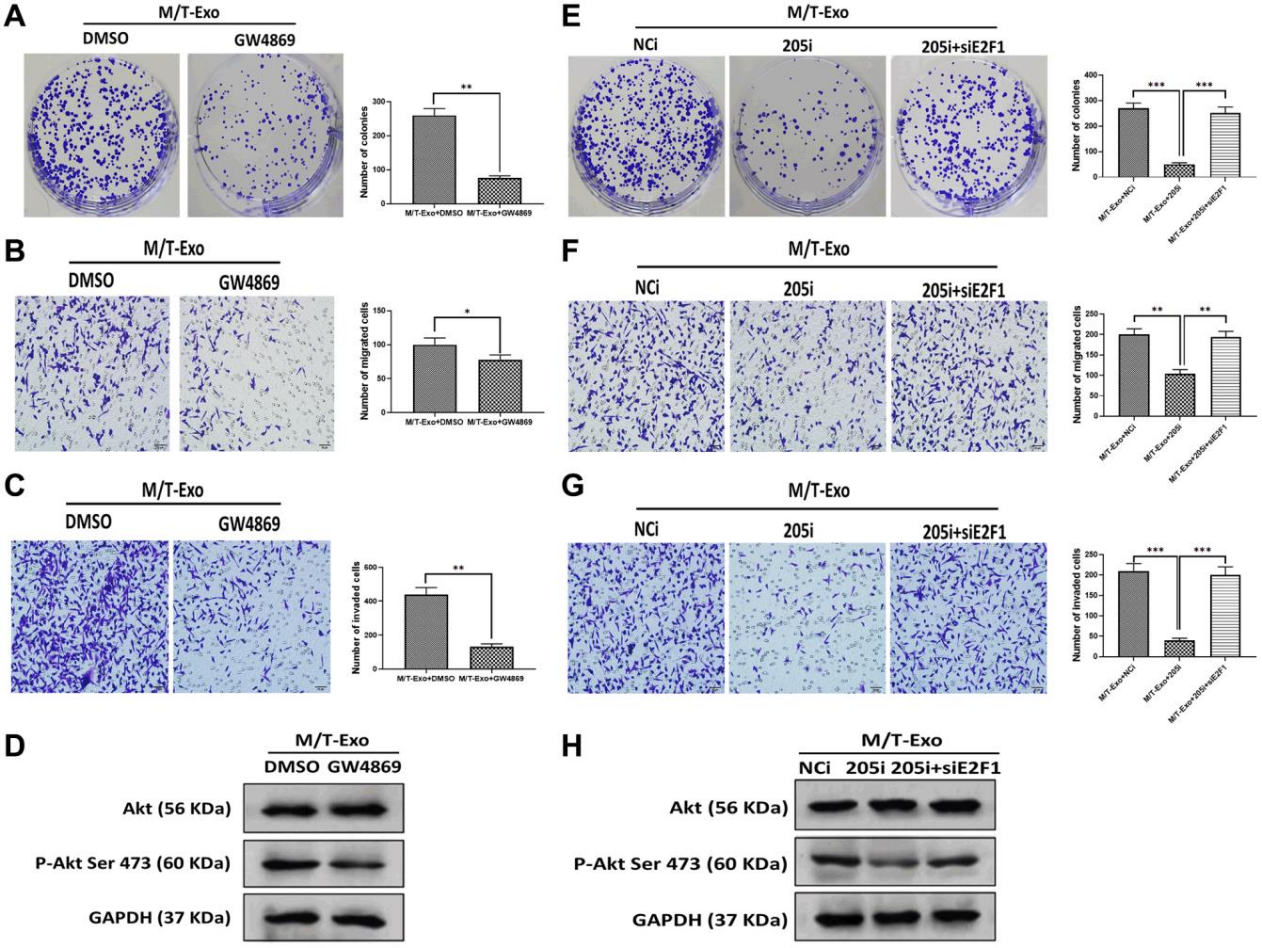
**M/T-Exo miRNA-205 affects breast cancer cells (BCCs) proliferation, migration, and invasion via targeting E2F1.** (**A**–**C**) The abilities of colony formation, migration, and invasion in M/T-Exo-cocultured BCCs treated with DMSO or GW4869. (**D**) The protein expression of Akt and the phosphorylation of Akt at Ser 473 (p-Akt Ser 473) in M/T-Exo-cocultured BCCs treated with DMSO or GW4869. (**E**–**G**) The abilities of colony formation, migration, and invasion in M/T-Exo-cocultured BCCs treated with the negative control miRNA-205 inhibitor (NCi), miRNA-205 inhibitor (205i), or the combination of 205i and E2F1 siRNA (siE2F1). (**H**) The protein expression of Akt and the phosphorylation of Akt at Ser 473 (p-Akt Ser 473) in M/T-Exo-cocultured BCCs treated with the negative control miRNA-205 inhibitor (NCi), miRNA-205 inhibitor (205i), or the combination of 205i and E2F1 siRNA (siE2F1). Values are means ± SD. ^*^P < 0.05, ^**^P < 0.01, ^***^P < 0.001. At least three replicates were available for analysis in each treatment group.

BCCs treated with NCi and M/T-Exo showed higher levels of proliferation, migration, and invasion (Figure 5A, 5B and 5C), in which the phosphorylation of Akt was elevated (Figure 5D). Additionally, BCCs cocultured with M/T cells and GW4869 showed inhibited proliferation, migration, and invasion abilities compared to the control group (Figure 6A, 6B and 6C). Meanwhile, the phosphorylation of Akt was decreased in BCCs treated with M/T cells and GW4869 (Figure 6D). Furthermore, the combination of M/T-Exo and 205i led to reduced levels of cell proliferation, migration, and invasion in BCCs (Figure 6E, 6F and 6G), accompanied with inhibited phosphorylation of Akt (Figure 6H). Together, exosomal miRNA-205 may enhance cell proliferation, migration, and invasion in BCCs via inhibiting Akt phosphorylation.

### Exosomal miRNA-205 promotes tamoxifen resistance and tumorigenesis of BC *in vivo*

**Figure 7:**
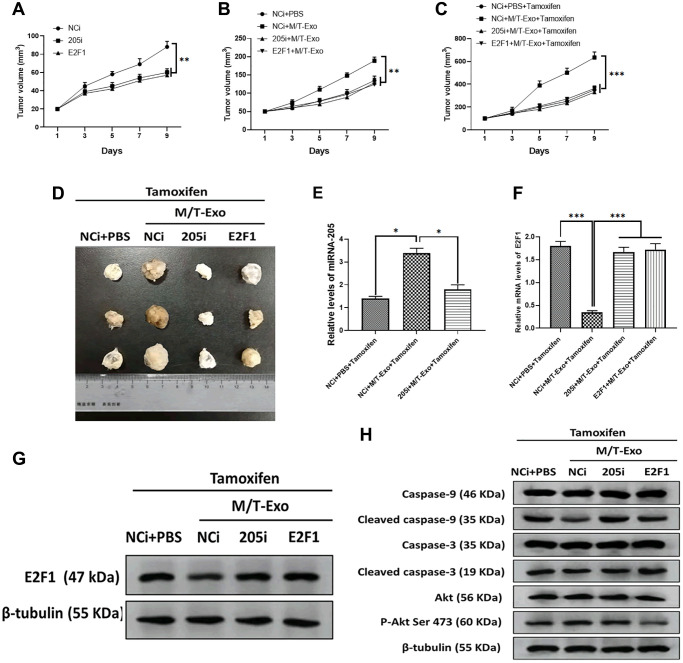
**M/T-Exo miRNA-205 enhances the tamoxifen resistance *in vivo*.** (**A**) Tumor growth curves of mice injected with the negative control miRNA-205 inhibitor (NCi), miRNA-205 inhibitor (205i), or lentiviral vector carrying E2F1 (E2F1). (**B**) Tumor growth curves of mice injected with M/T-Exo when the tumor size was around 50 mm^3^. (**C**) Tumor growth curves of mice injected with tamoxifen when the tumor size was around 100 mm^3^. (**D**). Representative images of the tumor. (**E**) After all treatments, the expression of miRNA-205 in tumors of mice on day 9 post-tamoxifen treatment. (**F**–**G**). The mRNA and protein expressions of E2F1 in tumors of mice on day 9 post-tamoxifen treatment. (**H**). The protein expressions of cleaved caspase-9 and caspase-3, Akt, and the phosphorylation of Akt at Ser 473 (p-Akt Ser 473) in tumors of mice on day 9 post-tamoxifen treatment. Values are means ± SD. ^*^P < 0.05, ^**^P < 0.01, ^***^P < 0.001. At least three replicates were available for analysis in each treatment group.

To further investigate the effect of exosomal miRNA-205 *in vivo*, nude mice were subcutaneously injected by BCCs treated with NCi, 205i, and E2F1, respectively. The results showed that the tumor of mice treated with NCi grew larger and faster than those of mice treated with 205i and E2F1, respectively (Figure 7A). Next, the NCi-treated mice were intratumorally treated with M/T-Exo where the tumors grew faster than those treated with PBS (Figure 7B). Furthermore, the tamoxifen treatment was applied to each group and the results showed that the mice treated with NCi and M/T-Exo exhibited greater tumor growth than those in the other groups (Figure 7C and 7D). These results suggest that M/T-Exo may promote tamoxifen resistance and tumorigenesis of BC *in vivo*. When testing whether the mechanism underlying exosomal miRNA-205 *in vivo* is same as those *in vitro*, the results indicated that the mice treated with the combination of NCi, M/T-Exo, and tamoxifen displayed higher expressions of miRNA-205 and lower mRNA and protein levels of E2F1 (Figure 7E, 7F and 7G). Correspondingly, the expressions of cleaved caspase-9 and caspase-3 and the phosphorylation of Akt was decreased and increased, respectively, in tumors of mice treated with the combination of NCi, M/T-Exo, and tamoxifen (Figure 7H). Therefore, these results suggest that exosomal miRNA-205 may promote tamoxifen resistance and tumorigenesis of BC *in vivo* via the caspase 9/3 and Akt signaling pathways.

## DISCUSSION

**Figure 8:**
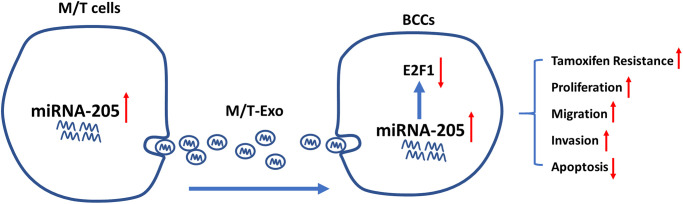
Graphical summary.

One common malignancy, BC, is also the second leading cause of tumor-associated mortality among females world-wide [38]. Although considerable progress and advances have been made in the diagnosis and treatment of BC, chemoresistance-induced metastatic recurrence is still a challenge for researchers in both basic and clinical settings [39]. Thus, overcoming chemoresistance-associated issues would significantly improve the therapeutic efficacy and survival of patients with BC. In the present study, we demonstrated that exosomal miRNA-205 could be transferred from chemoresistant M/T cells to BCCs to promote tamoxifen resistance through silencing E2F1 (Figure 8). Also, exosomal miRNA-205 derived from M/T cells was found to enhance proliferation, migration, and invasion abilities in BCCs. Furthermore, *in vivo* experiments also verified the effect of exosomal miRNA-205 in chemoresistance and tumorigenesis. Hence, we demonstrated that donor-originated exosomal miRNA-205 could be internalized by recipient cells, thus regulating its cellular activities in both *in vitro* and *in vivo*.

In this study, exosomes secreted from tamoxifen resistance M/T cells expressed higher levels of miRNA-205. Exosomes are extracellular vesicles (30 to 100 nm in diameter) and are secreted by various cell types through fusion of vesicular bodies with plasma membranes [40]. Exosomes can transfer functioning molecules, including miRNAs, mRNAs, proteins, and membrane components, between donor and recipient cells, which is regarded as a potential targeted delivery carrier of molecules to impact cellular activities [41]. Moreover, this study also found that BCCs cocultured with M/T-Exo exhibited more miRNA-205 expression and elevated tamoxifen resistance, indicating the essential roles of exosomal miRNA-205 in the modulation of chemoresistance in BCCs. Recently, exosomes also have been reported to exert key roles in the regulation of chemoresistance in various cancer types, especially miRNAs-loading exosomes. Yeung et al. reported that exosomal miRNA-21 derived from the omental stromal cells promote paclitaxel resistance in ovarian cancer [42]. Zheng et al. demonstrated that exosomal-carrying tumor-related macrophages, miRNA-21, confers cisplatin refractoriness in gastric cancer through upregulating the PI_3_K/Akt pathway via the inhibition of phosphatase and tensin homolog (PTEN) [43]. Thus, exosome-mediated intercellular communication may be a potential novel strategy for treating chemoresistance in cancers.

In addition to chemoresistance, exosomes-based communications have been demonstrated to be an effective way to regulate cancer development and progression. Exosomal miRNA-1246 enhances cell proliferation and invasion in BC through cyclin-G2 (CCNG2) signaling [44]. Long non-coding RNA MALAT1 delivered via exosomes facilitates BCCs proliferation and progression in BC [45]. Furthermore, circulating exosomal miRNA-96 enhances cell migration and proliferation in lung cancer through the LIM-domain only protein 7 (LMO7) pathway. In the present study, exosomal miRNA-205 promoted BCCs proliferation, migration, and invasion in BCCs via the Akt signaling pathway, suggesting an essential regulatory role of exosomal miRNAs in tumor cell activities. Collectively, exosome-carrying functioning molecules may be a promising strategy to manipulate the tumor progression, opening novel avenues to overcome cancers.

As a multifunctional factor, miRNA-205 has been reported to be involved in a variety of physiological and pathological processes, such as cell proliferation [46], angiogenesis [47], epithelial to mesenchymal transition [48], and the cellular oxidative stress response [49]. In the present study, the expression of miRNA-205 was observed to be upregulated in tamoxifen resistance M/T cells, which is consistent with the observations that upregulation of miRNA-205 is associated with drug-resistance in multiple cancer cells, including hepatocellular carcinoma [28], esophageal squamous cell carcinoma [50], and ovarian cancer [51]. Notably, the downregulation of miRNA-205 is also uncovered in several chemoresistance tumor cell types, such as BC [52], pancreatic cancer [27], as well as cholangiocarcinoma [53]. This opposite expression profile of miRNA-205 in chemoresistant tumor cells may result from the dynamic and aberrant miRNA expression trend and the complexity of the miRNA regulatory network [54]. Furthermore, the context-dependent regulatory mechanism in each tumor type or cancer cell line [55] and the complicated mechanism of chemoresistance [56, 57] may also contribute to the opposite expression pattern of miRNA-205. Thus, it may be worthwhile to validate the function of one certain molecule across various cancer types, even different cell lines derived from the same cancer.

To further determine the mechanism underlying exosomal miRNA-205-induced chemoresistance in BCCs, both mechanical and functional experiments were performed to investigate the target gene of miRNA-205. The results revealed that miRNA-205 directly binds to E2F1 and overexpression of E2F1 could reverse the effect of miRNA-205 on chemoresistance and tumorigenesis in BCCs. In addition, E2F1, a cellular transcription, belongs to the E2F family [46]. Also, E2F1 can modulate tumor inhibitor p53 as well as p73, facilitating apoptosis through activating multiple cell-death signaling pathways [58]. The upregulation of E2F1 is associated with elevated apoptosis levels of malignant cells in response to genotoxic treatment, thereby suppressing tumorigenesis [59, 60]. Moreover, E2F1 is shown to be a key regulator in chemotherapy-related apoptosis [59]. Previous studies suggest that the interaction between miRNA-205 and E2F1 plays an essential role in anti-tumor chemotherapy resistance [61, 62]. Together, E2F1 may be a promising biomarker to treat apoptosis-resistant tumors.

## MATERIALS AND METHODS

### Ethics statement

All subjects recruited in this study were informed before inclusion and written consents were given. This study was reviewed and approved by the Ethics Committee of Cangzhou Central Hospital (2017R-K1174). All animal involved experimental protocols and procedures were approved by the Institutional Animal Care and Use Committee of Cangzhou Central Hospital (CZ-208-R8743) and were performed based on the guidelines and regulation of the Management of Laboratory Animals published by the Ministry of Science and Technology of the People’s Republic of China.

### Cell culture

Breast tumor samples were collected from two patients who were diagnosed with BC and undergone BC surgical resection at the Cangzhou Central Hospital in April 2017 (35 and 46 years old). The detailed information of patients was summarized in Table 1. The diagnosis criteria were previously described [63] and the pathological stage, grade, and nodal status were evaluated by two experienced pathologists. Immediately after surgical resection, BCCs were isolated from tumor tissues according to a detailed protocol, as previously described [64]. Tamoxifen-resistant breast cancer cell line MCF-7/TAMR-1 (M/T) was purchased from MilliporeSigma (Catalog #: SCC101; Burlington, MA, USA). The BCCs and M/T cells were grown in appropriate growth mediums as previously described [64] and using the manufacturer’s instructions, respectively. Cells were passaged every 3 days and collected for subsequent experiments by trypsinization before reaching confluence.

**Table 1:** Clinicopathological characteristics of 2 patients with breast cancer.

**Characteristics**	**Patient 1**	**Patient 2**
Age (year)	35	46
Menopausal status	Premenopause	Premenopause
Tumor size (mm)	23.7	26.4
TNM stage	III	III
Pathological grade	III	III
Lymph node metastasis	Positive	Positive
Progesterone receptor	Positive	Positive
Her2 receptor	Positive	Positive
Subtype	mixed	mixed

### Exosome isolation and labeling

Exosome-free fetal bovine serum (FBS) (Catalog #: A2720801; Thermo Fisher Scientific, Waltham, MA, USA) was collected through 120,000 × g ultracentrifugation at 4°C for 6 hours. The BCCs and M/T cells (1 × 10^6^) were placed in the medium supplemented with 10% exosome-free FBS for 48 hours at 37°C in a humidified atmosphere with 5% CO_2_. Next, to collect the exosomes, 40 ml of conditioned mediums were taken from each cell line and the ExoQuick-TC Kit (Catalog #: EXOTC10A-1; System Bioscience, Palo Alto, CA, USA) was used. The morphology and diameter size of the exosomes were assessed by Morgagni 268D transmission electron microscopy (TEM; Philips, Bothell, WA, USA) according to a protocol previously described [65]. The PKH26 red fluorescent dye (Catalog #: PKH26GL; Sigma-Aldrich, St. Louis, MO, USA) was applied to label exosomes derived from M/T cells according to the manufacturer’s instructions.

### miRNA microarray

Total RNA was extracted from BCCs and M/T cells using the TRIzol reagent (Catalog #: 15596026; Invitrogen, Carlsbad, CA, USA). The concentration and quality of total RNA were examined by NanoDrop™ 2000 (Life Technologies; Carlsbad, CA, USA). The miRNA microarray and data analysis were performed as previously described [66, 67]. Briefly, the raw data were processed using the Affy package pair package in R language, including data filtering, log_2_ transformation, and normalization [68]. The average of three fluorescence signal intensities of each miRNA was normalized to 5sRNA. Then, normalized data were analyzed using the significance analysis of microarrays (SAM) algorithm [69]. The *t*-test analysis was performed between BCCs and M/T cells. Differentially expressed miRNAs were screened with a false discovery rate (FDR) corrected *p* <0.05 and |log_2_ fold-change (FC)|>2.

### Cell coculture system and GW4869 treatment

The coculture system of the exosome donor and recipient cells were modified as previously described [70]. The M/T cells were placed in the upper chamber of the system and BCCs were placed in the lower chamber. A membrane with 0.4 pores was used to separate the upper and lower chamber. The medium used in the coculture system was 10% exosome-free FBS. The GW4869 (Catalog #: D1692; Sigma-Aldrich, St. Louis, MO, USA) was applied to block exosome formation from M/T cells [71].

### Cell transfection

The miRNA-205 inhibitor (205i) and the corresponding negative control (NCi) were obtained from GenePharma Co., Ltd (Shanghai, China). The BCCs (1 × 10^6^/well) were transfected with 100 nM of 205i or NCi using the Lipofectamine™ 3000 Reagent (Catalog #: L3000015; Invitrogen, Carlsbad, CA, USA) according to the manufacturer’s instructions. Small interfering RNA (siRNA) specific for E2F1 (siE2F1) (sense, 5′-CCUGGAAACUGACCAUCAGTT-3′, antisense, 5′-CUGAUGGUCAGUUUCCAGGTT-3′) and E2F1-expressing plasmids (pcDNA3.1-E2F1, using the XhoI and EcoRI restriction sites) were obtained from GenePharma Co., Ltd (Shanghai, China). Co-transfection of 50 nM 205i and 50 nM siE2F1 was performed according to the manufacturer’s instructions using the Lipofectamine™ 3000 Reagent (Catalog #: L3000015; Invitrogen, Carlsbad, CA, USA). After 24 hours of transfection, transfected cells were used for subsequent experiments.

### Luciferase reporter assays

The BCCs that were treated with M/T-Exo or control-miRNA were transfected with plasmids carrying the wild or mutant miRNA binding sequence in 3′UTR of E2F1 using the Lipofectamine™ 3000 Transfection Reagent (Catalog #: L3000015; Invitrogen, Waltham, MA, USA) according to the manufacturer’s instructions. After 24 hours of transfection, relative luciferase activities were quantified using the Luciferase Reporter Assay Substrate Kit (Catalog #: ab228530; Abcam, Cambridge, MA, USA).

### Quantitative real-time PCR (qRT-PCR)

Total RNA was extracted from exosomes, cells, and tumor tissues of xenograft mice using the TRIzol reagent (Catalog #: 15596026; Invitrogen, Carlsbad, CA, USA). The qRT-PCR reactions were performed as previously described [72]. The relative expression of miRNA-205 was normalized to U6 and was calculated using the 2^−ΔΔCT^ method [73]. Table 2 lists the primers used in this study.

**Table 2:** Primer information.

**Gene name**	**Primer sequence**
RNU6 (F)	5′-CGCTTCGGCAGCACATATACTAAAATTGGAAC-3′
RNU6 (R)	5′-GCTTCACGAATTTGCGTGTCATCCTTGC-3′
miR-181a-5p (F)	5′-GAACATTCAACGCTGTCGGTG-3′
miR-181a-5p (R)	5′-ATCCAGTGCAGGGTCCGAGGTA-3′
miR-21-3p (F)	5′-CGCGCCAACACCAGTCGATG-3′
miR-21-3p (R)	5′-GTGCAGGGTCCGAGGT-3′
miR-125b (F)	5′-GGCAACCTTGCGACTATAACCA-3′
miR-125b (R)	5′-GTTTCCTCTCCCTGAGACCCTA-3′
miR-200c (F)	5′-AGCGGTAATACTGCCGGGTA-3′
miR-200c (R)	5′-GTGCAGGGTCCGAGGT-3′
miR-205 (F)	5′-CGTCCAACATTCCACCG-3′
miR-205 (R)	5′-GTGCAGGGTCCGAGGT-3′
miR-99a (F)	5′-GTTGGATCCTATTAATAGGGGGCCCATGCAAGAT-3′
miR-99a (R)	5′-GTTGGATCCTATTAATAGGGGGCCCATGCAAGAT-3′

### Western blots

Total protein isolation of exosomes, cells, and tumor tissues of xenograft mice and western blots were performed as previously described [6]. The primary antibodies against caspase-9 (1:500; Catalog #: ab25758) and caspase-3 (1:500; Catalog #: ab184787) and cleaved caspase-9 (1:500; Catalog #: ab2324)) and caspase-3 (1:500; Catalog #: ab2302) were obtained from Abcam (Cambridge, MA, USA). The primary antibodies against E2F1 (1:500; Catalog #: sc-251), CD63 (1:500; Catalog #: sc-5275), CD81 (1:500; Catalog #: sc-23962), HSP70 (1:500; Catalog #: sc-32239), Akt (1:500; Catalog #: sc-56878), p-Akt Ser 473 (1:500; Catalog #: sc-293125), β-tubulin (1:1000; Catalog #: sc-166729), and GAPDH (1:1000; Catalog #: sc-47724) were obtained from Santa Cruz (Santa Cruz, Shanghai, China). Optical densities of the band were quantified using the Uvitec Alliance software (Eppendorf, Hamburg, Germany).

### Quantification of apoptosis

The Annexin V-APC Assay Kit (Catalog #: ab236215; Cambridge, MA, USA) and flow cytometry assay were used to quantify cell apoptosis according to the manufacturer’s instructions.

### CCK-8, colony formation, migration, and invasion assays

Evaluation for cell proliferation, colony formation, migration, and invasion were performed as previously described [74]. Cell proliferation was determined using Cell Counting Kit-8 (CCK-8) (Catalog #: ab228554; Abcam, Cambridge, MA, USA). Crystal violet used to stain cell colony was purchased from Santa Cruz (Catalog #: CAS 548-62-9; Santa Cruz, Shanghai, China). Migration and invasion assays were performed using transwell chambers (Catalog #: ECM550 and ECM508; Sigma-Aldrich, St. Louis, MO, USA) according to the manufacturer’s instructions.

### Xenograft mouse model

Twelve male BALB/c nude mice (6 weeks old) were obtained from Jackson Labs (Bar Harbor, ME, USA). Transfected BCCs (1 × 10^6^) in 100 μl PBS and Matrigel were subcutaneously injected into each mouse. Mice were randomly divided into four groups (*n* = 3): mice in group 1 and 2 were injected with NCi-treated cells, mice in group 3 were injected with 205i-treated cells, and mice in group 4 were injected with E2F1-treated cells. Tumor growth was monitored daily and tumor volume (V) was evaluated by measuring the length (L) and width (W) with a caliper and calculated using the formula: V = (L × W^2^) × 0.5. When the tumor tissue volume was around 50 mm^3^, the mice in group 2, 3, and 4 were intratumorally treated with M/T-Exo (40 μg) three times a week. Once the tumor tissue volume was around 100 mm^3^, all mice were treated with tamoxifen (3 mg/kg) through the tail vein three times a week for two weeks and then were sacrificed. The tumor tissues were isolated and immediately stored in –80°C for subsequent experiments.

### Statistical analysis

In this study, data were presented as means ± SD from at least 3 independent replicates. The statistical analyses were completed using the SPSS 17.0 software (SPSS, Chicago, USA). Two-tailed Student’s *t*-test was applied to analyze statistical differences between two experimental groups. One-way analysis of variance (ANOVA) with post-hoc test was applied to analyze statistical differences between three or more experimental groups. *P* < 0.05 was considered statistically significant.

## CONCLUSIONS

In conclusion, the results suggest that exosomal miRNA-205 could promote tamoxifen resistance and tumorigenesis in BC through targeting E2F1 *in vivo* and *in vitro* and that exosomes-mediated transfer of functioning molecules may provide novel insight into developing therapeutic strategies for BC.
